# Einfluss der COVID-19-Pandemie auf Anzahl und Dauer der von ambulanten Pflegediensten erbrachten Pflegeberatungen nach § 37 Abs. 3 SGB XI in Bayern

**DOI:** 10.1007/s16024-021-00358-8

**Published:** 2021-11-02

**Authors:** Jörg Hallensleben, Claudia Wöhler

**Affiliations:** 1 APOLLON Hochschule der Gesundheitswirtschaft GmbH, Universitätsallee 18, 28359 Bremen, Deutschland; 2 BARMER Landesvertretung Bayern, Landsberger Str. 187, 80687 München, Deutschland

**Keywords:** Pflegeberatung, Beratungsdauer, Pflegeberatungsverpflichtung, COVID-19-Pandemie, Bayern, Care counseling, Nursing consultations, COVID-19 pandemic, German long-term care insurance, Home health care

## Abstract

**Hintergrund:**

Als Reaktion auf die COVID-19-Pandemie hat der deutsche Gesetzgeber die Rahmenbedingungen für die Pflegeberatung nach § 37 Abs. 3 SGB XI geändert. Die hauptsächlich durch ambulante Pflegedienste erbrachte Pflegeberatung muss (befristet bis zum 31.12.2021) nicht mehr in der Häuslichkeit der Pflegebedürftigen erfolgen, sondern ist auch per Telefon oder online möglich. In 2020 war zudem die bestehende Verpflichtung zur Inanspruchnahme einer Pflegeberatung für Pflegegeldbeziehende für einige Monate ausgesetzt.

**Ziel:**

Beschrieben wird der Einfluss der COVID-19-Pandemie auf Anzahl und Dauer der von ambulanten Pflegediensten erbrachten Pflegeberatungen nach § 37 Abs. 3 SGB XI. Ein besonderes Augenmerk liegt auf der Frage, wie sich die Aussetzung der Beratungsverpflichtung auf die Inanspruchnahme von Pflegeberatungen ausgewirkt hat.

**Methode:**

Sekundäranalyse von über 43.000 Datensätzen der BARMER Pflegekasse in Bayern, die ursprünglich zum Zwecke der Abrechnung gesammelt und gespeichert wurden. Auswertung in erster Linie mittels Zeitreihenanalysen und anderer deskriptiver Statistiken. Zusätzlich wurde die Korrelation zwischen der wöchentlichen Anzahl der Pflegeberatungen und den dem Robert Koch-Institut gemeldeten COVID-19-Fälle in Bayern ermittelt.

**Ergebnisse:**

Ende März bis Mitte April 2020 verringerte sich die Zahl der Beratungen gegenüber Februar 2020 um fast 80 %. Für diesen Einbruch der Beratungszahlen war die Sorge vor einer Ansteckung mit SARS-CoV-2 entscheidend. Ermöglicht wurde der Rückgang aber zusätzlich durch die am 27.03.2020 beschlossene Aussetzung der Beratungspflicht. Die Aussetzung der Beratungspflicht allein hat in Bayern zu einem Rückgang zwischen 44 und 49 % geführt. Die vorliegenden Daten zeigen ferner, dass telefonische Pflegeberatungen im Durchschnitt kürzer sind als Präsenzberatungen.

**Schlussfolgerungen:**

Die vorliegenden Daten deuten darauf hin, dass nur rund die Hälfte der Pflegeberatungen von Pflegebedürftigen in Anspruch genommen würde, wenn die in § 37 Abs. 3 verankerte Beratungsverpflichtung dauerhaft wegfiele. Außerdem enthalten sie Hinweise darauf, dass Beratungen bei einer Abrechnung nach Zeit im Durchschnitt kürzer sind als bei einer Abrechnung mittels Einsatzpauschalen.

## Abstract (en)

**Background:**

In response to the COVID-19 pandemic, the German legislator has changed the framework conditions for care counselling (provided mainly by outpatient care services) in the German long-term care insurance (§ 37 (3) SGB XI). The care counselling no longer has to be provided in the home of the patients but is also possible by telephone or online. In 2020, the existing obligation for family members and other informal caregivers to make use of a care counselling service was suspended for a few months.

**Aim:**

The aim of this study was first of all to describe the influence of the COVID-19 pandemic on the number and duration of care counselling by outpatient care services in Bavaria, Germany. Particular attention was paid to the following question: what impact did the suspension of the obligation to make use of care counselling have on the utilization of care counselling?

**Method:**

Secondary analysis of more than 43,000 data sets of the BARMER Nursing Insurance in Bavaria. Evaluation primarily by means of time series analyses and other descriptive statistics. In addition, the correlation between the weekly number of care counselling consultations and the cases of COVID-19 in Bavaria was calculated.

**Results:**

At the end of March and in April 2020 the number of consultations decreased by nearly 80% compared to February 2020. The concern about contagion with SARS-CoV-2 was crucial to this slump in the number of counselling visits; however, the reduction was also made possible by the suspension of the obligation to provide care counselling, which was decided on 27 March 2020. The suspension of the obligation to provide advice alone led to a decrease of between 44% and 49% in Bavaria. The available data also indicate that telephone consultations are shorter than face-to-face consultations.

**Conclusion:**

The available data suggest that only about half of the care consultations would be used by users if the obligation to make use of care counselling—as laid down in § 37 (3) SGB XI—were permanently eliminated. In addition, they contain clear indications that remuneration after time (as practised in Bavaria) has led to an extension of the consultation times.

## Hintergrund

Mit 90,6 % wird die Mehrzahl der Pflegeberatungen nach § 37 Abs. 3 SGB XI (Sozialgesetzbuch Elf) werden durch ambulante Pflegedienste durchgeführt (Wolff et al. 2020, S. 181). Diese Form der Pflegeberatung war bis zum 01.01.2017 (dem Inkrafttreten der durch das Zweite Pflegestärkungsgesetz – PSG II vorgenommenen Änderungen am SGB XI) an den Bezug von Pflegegeld gekoppelt. Bis heute ist der ausschließliche Bezug von Pflegegeld an die Bedingung geknüpft, dass die erforderlichen körperbezogenen Pflegemaßnahmen, pflegerischen Betreuungsmaßnahmen sowie Hilfen bei der Haushaltsführung in geeigneter Weise sichergestellt werden – d. h., auch ohne die durch ambulante Pflegedienste angebotenen Pflegesachleistungen nach § 36 SGB XI (Wahl 2018, S. 293). Pflegegeldbeziehende, die ihre Versorgung informell sicherstellen, müssen daher – zwecks Qualitätssicherung – eine Pflegeberatung nach § 37 Abs 3. SGB XI in Anspruch nehmen. Ruft die pflegebedürftige Person diese Beratung nicht ab, haben die Pflegekasse oder das private Versicherungsunternehmen das Pflegegeld angemessen zu kürzen und im Wiederholungsfall zu entziehen (§ 37 Abs. 6 SGB XI). Während für diejenigen Pflegebedürftigen, die ausschließlich Pflegegeld beziehen, bis zu 4 Pflegeberatungsbesuche im Jahr obligatorisch sind, haben Pflegebedürftige mit Pflegegrad 1 sowie Pflegebedürftige der Pflegegrade 2 bis 5, die Pflegesachleistungen nach § 36 SGB XI in Anspruch nehmen, ein Recht auf bis zu 2 Pflegeberatungsbesuche/Jahr. Der Beratungsbesuch ist gegenüber der Pflegekasse nachzuweisen (GKV-Spitzenverband 2020, S. 165).

Nach dem heutigen Wortlaut des SGB XI dient die Pflegeberatung der „Sicherung der Qualität der häuslichen Pflege“ sowie der „regelmäßigen Hilfestellung und praktischen pflegefachlichen Unterstützung“ der häuslich Pflegenden (§ 37 Abs. 3 Satz 2 SGB XI). Den 37.3-Pflegeberatungen kommt eine zentrale Funktion für die Sicherstellung der häuslichen Pflege zu (GQP 2019). Sie erreichen gerade wegen des größtenteils obligatorischen Charakters mehr von Überforderung bedrohte Familien als die Pflegeberatung der Krankenkassen nach § 7a SGB XI oder andere Beratungsformate. Von daher wird ihnen die präventive Rolle eines „kontinuierlichen Türöffners“ zugeschrieben (Angele und Calero 2019, S. 326).

### Forschungsfragen und Hypothesen zu den Auswirkungen der COVID-19-Pandemie auf die Inanspruchnahme der Pflegeberatung

Wie fast alle anderen Bereiche des öffentlichen und privaten Lebens (Gärtner et al. 2021) wurde auch die Pflegeberatung von der COVID-19-Pandemie massiv betroffen. Am 27.01.2020 war bei dem Automobilzulieferer Webasto ein noch eingrenzbarer Ausbruch der Krankheit erfolgt. Die eigentliche erste Welle der COVID-19-Pandemie entwickelte sich – zunächst noch mit Einzelfällen – ab dem 27. Februar; sie erreichte im letzten Märzdrittel ihren Höhepunkt und ebbte bis Ende April/Anfang Mai ab (RKI 2020, 2021a). Korrespondierend zu dieser Entwicklung ergriff die bayerische Staatsregierung verschiedene Gegenmaßnahmen, u. a. die am 21.03.2020 beschlossenen weitreichenden Ausgangsbeschränkungen. Im Laufe des Monats Mai wurde ein Großteil dieser Maßnahmen dann wieder schrittweise zurückgenommen oder wenigstens gelockert.

Zu den zahlreichen Ausnahmeregelungen, die bundesweit unter dem Eindruck der COVID-19-Pandemie erlassen wurden, gehörte auch der § 148 SGB XI. Dieser wurde durch Artikel 4 des COVID-19-Krankenhausentlastungsgesetzes vom 27.03.2020 in das Elfte Sozialgesetzbuch eingefügt. In der ersten Fassung des § 148 SGB XI wurde die Verpflichtung zur Inanspruchnahme einer Pflegeberatung nach § 37 Abs. 3 SGB XI bis zum 30.09.2020 ausgesetzt.

Trotz pausierender Beratungsverpflichtung hatten Pflegebedürftige (und deren Angehörige) weiterhin einen Rechtsanspruch auf fakultative Pflegeberatungen. Soweit nicht aufgrund einer lokal begrenzten Lockdown-Regelung oder einer Quarantäne im Einzelfall jede Begegnung mit einer Person eines anderen Haushalts untersagt war, konnten Beratungen in der eigenen Häuslichkeit durchgeführt werden – natürlich unter Beachtung der gebotenen Schutzmaßnahmen, wie Abstandswahrung, Händedesinfektion und Tragen von Mund-Nasen-Schutz. Da jedoch auch mit Einhaltung von Sicherheitsmaßnahmen ein Restrisiko auf Ansteckung mit dem SARS-CoV-2 nicht gänzlich auszuschließen war, drohte ein massiver Einbruch bei den Beratungszahlen.

Um auch solchen Pflegebedürftigen eine 37.3-Pflegeberatung zu ermöglichen, die aus Angst vor einer Ansteckung Besuche in der Häuslichkeit ablehnten, ließ der GKV-Spitzenverband bereits am 18.03.2020 in einem Rundschreiben die Möglichkeit zu, Beratungen nach § 37 Abs. 3 SGB XI ausnahmsweise auch telefonisch durchzuführen. Diese Möglichkeit wurde am 15.04.2020 in einem weiteren Rundschreiben bekräftigt (GKV-Spitzenverband 2020a) und fand einige Monate später sinngemäß Eingang in das Elfte Sozialgesetzbuch. Nach der inzwischen überholten Fassung des § 148 SGB XI (hergestellt durch Artikel 3 des Gesetzes zur Verbesserung der Gesundheitsversorgung und Pflege, GPVG, vom 22.12.2020 und bis zum 31.12.2021 verlängert durch Art 4 Nr. 6 des Gesetzes zur Fortgeltung der die epidemische Lage von nationaler Tragweite betreffenden Regelungen) erfolgte die von den Pflegebedürftigen abzurufende Beratung „telefonisch, digital oder per Videokonferenz, wenn die oder der Pflegebedürftige dies wünscht“. Nach einer Befragung von ambulanten Pflegediensten (*N* = 599) durch Wolf-Ostermann, Rothgang et al. (2020, S. 49) wurden Videokonferenzen allerdings nur selten für Kontakte mit pflegebedürftigen Menschen (3 %) und ihren Angehörigen (5 %) genutzt; die Beratungen fanden also – wenn nicht in Präsenz – dann in erster Linie telefonisch statt.

Obwohl sich bei den Pflegekassen spätestens im Laufe des Monats April die Einsicht durchsetzte, auch telefonische Pflegeberatungen zu akzeptieren, dürfte die Mehrzahl der Pflegebedürftigen bis weit in den Mai keine Kenntnis von dieser Information gehabt haben; sie werden also weiterhin davon ausgegangen sein, dass eine Pflegeberatung nur in der eigenen Häuslichkeit möglich wäre. Da insoweit von Ende März bis Mitte Mai die Aussetzung der Beratungspflicht sowie die Sorge vor einer Ansteckung mit COVID-19 darin zusammenwirkten, die Nachfrage nach Pflegeberatungen zu dämpfen, stellte (und stellt) sich nicht die Frage, *ob*, sondern *in welchem Ausmaß die Pflegeberatungen in dieser Zeit zurückgegangen sind* (= *Forschungsfrage* F1). Erwartet wurde (*Forschungshypothese *H1) ein *sehr starker Rückgang der 37.3-Pflegeberatungen in diesem Zeitraum, mindestens eine Halbierung im Vergleich zu den Monaten vor dem COVID-19-Ausbruch*.

*Forschungsfrage* F2 lautete: Inwieweit *führt(e) die Aussetzung der Beratungspflicht – unabhängig von der Angst vor einer Infektion mit SARS-CoV-2 – zu einem Rückgang der Nachfrage nach 37.3-Pflegeberatungen?*

Kurz zum Hintergrund dieser Frage. Nach den vor der Pandemie durchgeführten Studien scheinen die Pflegegeldbeziehenden zwar recht zufrieden mit der 37.3-Pflegeberatung (gewesen) zu sein.

- So hatten, laut einer Befragung durch das IGES-Institut (Wolff et al. 2020), 90,7 % der Pflegegeldbeziehenden (*n* = 2038) „das Gefühl, dass sich die Beratungsperson ausreichend Zeit für den letzten Beratungsbesuch genommen hat“, und die aufkommenden Fragen wurden, nach Ansicht von 91,3 % der Befragten (*n* = 2044), meistens beantwortet (Wolff et al. 2020, S. 185).
- Weit überwiegend zufrieden mit der Pflegeberatung durch ambulante Pflegedienste äußerten sich auch die 1862 Befragten einer BARMER-Versichertenbefragung im Dezember 2017 (Rothgang und Müller 2018, S. 144).

Dennoch gab es immer schon Grund zu der Annahme, dass ein Teil der zur 37.3-Pflegeberatung verpflichteten Pflegegeldbeziehenden für sich keinen ausreichend großen Nutzen in einer Beratung sehen würde, um freiwillig eine solche in Anspruch zu nehmen (und sich sogar noch angesichts des Ansteckungsrisikos proaktiv um Termine zu kümmern). Die aufgrund der COVID-19-Pandemie erlassene gesetzliche Ausnahmeregelung (§ 148 SGB XI a. F.) hat erstmals die Möglichkeit eröffnet, diese Annahme empirisch zu quantifizieren. Erwartet wurde (*Forschungshypothese *H2) *ein starker Rückgang der 37.3-Pflegeberatungen während der gesamten Geltungsdauer der Ausnahmeregelung* (aber weniger stark als in der Zeit von Ende März bis Mitte April, die zusätzlich von einer sehr starken Sorge vor einer Ansteckung mit SARS-CoV‑2 geprägt gewesen ist).

### Forschungsfragen und Hypothesen zur Dauer der Pflegeberatungen

Die Dauer einer Pflegeberatung ist von beträchtlichem Interesse, da sich im Faktor „Zeit“ die qualitative und die finanzielle Perspektive verbinden (Geld ≈ Zeit ≈ Qualität). Da der Erfolg bzw. das Gelingen einer Beratung von vielen situativen, prozessualen und strukturellen Faktoren abhängt (nicht zuletzt von der Qualifikation der Beraterin oder des Beraters; Palesch 2019, S. 91 ff.), existiert sicherlich keine Gesetzmäßigkeit, wie „je länger die Dauer des Beratungsgesprächs, desto besser die Beratungsprozessqualität“. Wenn jedoch die Pflegeberatung mehr sein soll als ein Ankreuzen von Feldern auf dem Standardformular, nämlich eine umfassende Eruierung der Situation, ein Herausfinden der Wünsche und Ziele sowie das Entwickeln von Lösungen, dann ist das nicht in wenigen Minuten zu erledigen.

Mit Blick auf die Dauer der Pflegeberatungen wird v. a. die nachfolgende Forschungsfrage untersucht: (F3) *Sind telefonisch durchgeführte Pflegeberatungen im arithmetischen Mittel kürzer als Beratungen in der Häuslichkeit der zu beratenden Personen?*

Aus dieser Forschungsfrage lässt sich folgende allgemeine *Hypothese *ableiten: Je mehr Pflegeberatungen in einer Periode per Telefon durchgeführt werden, desto kürzer sind die Pflegeberatungen in dieser Periode im Durchschnitt. Stärker auf die zur Verfügung stehenden Daten ausgerichtet und insofern operationalisiert, lautet die Hypothese H3: *In dem Maße, wie Pflegedienste (und deren Beratungskunden) Kenntnis von der telefonischen Beratungsoption erhalten haben, hat sich die durchschnittliche Beratungsdauer im Monat verkürzt (deutliche Verkürzung von März auf April, weiter verkürzend im Mai und Juni).*

Schließlich soll auch noch eine Forschungsfrage beantwortet werden, die nicht unmittelbar mit COVID-19 zusammenhängt, nämlich: (F4) *Wie wirken sich die Art und die Höhe der Vergütung für ambulante Pflegeberatung nach § 37 Abs. 3 SGB XI auf die Dauer der Pflegeberatung aus?*

Zum Verständnis dieser Frage sind folgende Hintergrundinformationen nötig. Vor gut 10 Jahren nahmen die Besuche im Durchschnitt rund 30 min in Anspruch; wobei der erste Besuch in einem Haushalt im Regelfall deutlich länger dauerte als Folgebesuche und im Übrigen auch größere Unterschiede zwischen den Anbietern und natürlich den Beratungskunden existierten (Büscher et al. 2010, S. 24 f., 42). Die bis 2019 in § 37 Abs. 3 SGB XI auf eher niedrigem Niveau festgelegte Vergütung für die Beratungsbesuche machte längere Besuche für die Pflegedienste unwirtschaftlich. Im Jahr 2018 befand die Bundesregierung, dass die gesetzlichen Vergütungssätze in Höhe von 33 € für einen Beratungsbesuch bei Pflegestufe III bzw. 23 € für Beratungsbesuche bei Pflegestufen I und II (§ 37 Abs. 3 SGB XI a. F.) nicht mehr ausreichend wären, um die geforderte fachlich anspruchsvolle Beratung zu realisieren (PpSG – Entwurf, S. 98 f.). Seit dem Inkrafttreten der entsprechenden Regelungen aus dem Pflegepersonal-Stärkungsgesetz (PpSG) am 01.01.2019 werden die Vergütungen nicht mehr vom Gesetzgeber festgelegt, sondern sie werden nach den Grundsätzen des ambulanten Vergütungsrechts gemäß § 89 Abs. 1 und 3 SGB XI zwischen den Pflegekassen und den Pflegediensten bzw. deren Verbänden auf Landesebene ausgehandelt (§ 37 Abs. 3 Satz 3 SGB XI).

In den meisten Bundesländern wird heute eine auf Landesebene vereinbarte Punktzahl mit dem individuellen Punktwert des Pflegedienstes multipliziert. Beispiel: In Nordrhein-Westfalen beträgt die landesweite Punktzahl für den Leistungskomplex LK 17 „Beratungsbesuch nach § 37,3 SGB XI“ 1350 Punkte. Bei einem einrichtungsindividuell vereinbarten Punktwert von „0,040 €“ ergäbe sich daraus eine Vergütung von 54,80 € für den Leistungskomplex. Der Punktwert könnte aber durchaus deutlich höher liegen; beim „Caritasverband für die Stadt Köln e. V. Sozialstation Köln-Deutz/Kalk“ liegt er z. B. bei 0,067, woraus sich eine Vergütung von 90,46 € ergibt (Angaben aus www.pflegelotse.de am 22.03.2021). Grundsätzlich anders ermittelt wird die Vergütung eines Beratungsbesuches in Bayern. Die Vergütung erfolgt hier nicht pauschal, sondern nach Zeit; je angefangene 5 min kann jeder Pflegedienst 4,20 € abrechnen. Maximal sind insgesamt 75 min je Beratungseinsatz und damit 63,00 € abrechenbar (§ 6 Abs. 5 Vertrag gemäß § 89 SGB XI).

Festzuhalten ist erstens, dass sich die Vergütung der Beratungsbesuche in Bayern seit der in 2019 erfolgten PpSG-Gesetzesreform aus Sicht der Dienste deutlich verbessert hat, und zweitens, dass seither die Anreize so gesetzt sind, dass Pflegedienste mutmaßlich eher lange Beratungsgespräche führen als kurze.

Vor diesem Hintergrund lautet die *Hypothese* H4.1: *Die durchschnittliche Dauer eines 37.3-Pflegeberatungsbesuchs war nach Einführung des neuen Abrechnungssystems in Bayern – operationalisiert: in den Monaten August 2019 bis Februar 2020 – deutlich länger als davor. *Weiter wurde folgende *Hypothese* H4.2 aufgestellt: *In den Monaten seit dem Pandemieausbruch hat sich zwar die durchschnittliche Dauer eines 37.3-Pflegeberatungsbesuchs verkürzt (verglichen mit Monaten August 2019 bis Februar 2020), sie ist aber immer noch länger als vor Einführung des neuen Abrechnungssystems.*

## Methode

Datengrundlage der vorliegenden Studie sind routinemäßig zum Zwecke der Abrechnung erfasste Daten der BARMER Pflegekasse im Bundesland Bayern. Es handelt sich damit um eine Sekundäranalyse vorliegender Daten. Um den datenrechtlichen Bestimmungen sowie den Leitlinien und Empfehlungen für die Gute Praxis Sekundärdatenanalyse (AGENS 2014) zu entsprechen, wurden für die vorliegende Studie gespeicherte Routinedaten in anonymisierter Form in eine Excel-Datei überführt. Übergeben an Excel (Microsoft Corporation, Redmond, WA, USA) wurden nur Daten der Parameter „Datum der Zahlung“ und „Zahlbetrag“ – d. h. nicht direkt die Dauer des Einsatzes und auch keine kennzeichnenden Merkmale der Versicherten oder Pflegedienste. Aufgrund der oben skizzierten besonderen bayerischen Abrechnungslogik lässt sich aber aus dem jeweiligen Zahlbetrag für einen Beratungseinsatz mittels Division durch den bekannten Preis pro angefangene 5 min ohne Weiteres die Anzahl der von den Pflegediensten veranschlagten 5‑min-Einheiten ermitteln und daraus wiederum die hier interessierende Dauer des Beratungseinsatzes in Minuten.

Warum werden nicht direkt die von den Pflegediensten auf dem Beratungsprotokoll gemachten Angaben hinsichtlich Beginn und Ende des Beratungseinsatzes verwendet? Grund hierfür ist, dass die mit dem bundesweit einheitlichen Formular „Nachweis über einen Beratungsbesuch nach § 37 Abs. 3 SGB XI“ (GKV-Spitzenverband, o.J.) erstellten Beratungsprotokolle nicht standardmäßig maschinell ausgelesen werden und von daher keine maschinelle Gesamtdatenlieferung für den auszuwertenden Zeitraum verfügbar gewesen ist.

Grundlage der hier durchgeführten Sekundäranalyse sind über 43.000 Datensätze der BARMER Pflegekasse aus den Monaten August 2019 bis Dezember 2020. Datensätze mit einer errechneten „Beratungsdauer“ von weniger als 5 min wurden ausgeschlossen, da davon auszugehen ist, dass unterhalb dieser Schwelle keine Beratung stattgefunden haben konnte. Selbst eine – nicht fachgerechte – telefonische Kurzintervention („Geht es Ihnen im Wesentlichen immer noch so wie bei meinem letzten Besuch? Kann ich etwas für Sie tun?“) wäre mit Begrüßung und Verabschiedung kaum unter 5 min zu bewerkstelligen. Daher wird hier davon ausgegangen, dass die Pflegeberaterinnen und -berater bei kürzeren Gesprächen (sei es telefonisch, sei es vor Ort) nur über das Aussetzen der Beratungspflicht informiert und/oder Termine abgesprochen haben.

Die gewonnenen Daten sind mithilfe deskriptiver Statistik (Excel) beschrieben und analysiert. Im Einzelnen ausgewertet sind: Häufigkeiten, Mittelwerte (arithm. Mittelwert, Median), Minimal- und Maximalwerte, 1. und 3. Quartile sowie die Standardabweichungen. Zusätzlich berechnet ist die Korrelation zwischen der wöchentlichen Anzahl der Pflegeberatungen in Bayern und den dem Robert Koch-Institut gemeldeten 7‑Tage-Inzidenzen in Bayern.

Die Zeitreihen sind visualisiert. Auf die ursprünglich beabsichtigte Formulierung mathematischer Modelle der Zeitreihen und deren Schätzung mittels Regressionsanalyse (Backhaus et al. 2015) wurde verzichtet, da sich die Zeiträume, in denen die zu untersuchenden Faktoren wirksam waren oder nicht, nicht scharf voneinander abgrenzen lassen.

## Ergebnisse

### Repräsentativität der Stichprobe

Die Vollerhebung der von der BARMER Pflegekasse abgerechneten Pflegeberatungen kann als repräsentative Stichprobe für die von ambulanten Pflegediensten durchgeführten Pflegeberatungen nach § 37 Abs. 3 SGB XI für Bayern angesehen werden.

Ende 2017 lebten in Bayern 283.390 pflegebedürftige Menschen in ihrer eigenen Häuslichkeit. 185.799 davon waren zur Inanspruchnahme von Pflegeberatung verpflichtet; davon 153.831 als (ausschließlich) Pflegegeldbeziehende mit Pflegegrad 2 oder 3 zu 2 Beratungseinsätzen sowie weitere 31.968 mit Pflegegrad 4 oder 5 zu 4 Beratungseinsätzen/Jahr (Bayerisches Landesamt für Statistik 2021). Insgesamt wären damit 435.534 Pflichtberatungen durchzuführen gewesen. Da allerdings aus der Untersuchung von Wolff et al. (2020, S. 174) bekannt ist, dass Pflegegeldbeziehende im Mittel nur 1,5 Beratungen nach § 37 Abs. 3 SGB XI im Jahr in Anspruch nehmen, ist eher von rund 280.000 Pflichtberatungseinsätzen im Jahr auszugehen. Weitere 97.591 Personen hatten ein Recht auf Inanspruchnahme von bis zu 2 Pflegeberatungen/Jahr – nämlich 6130 Personen mit Pflegegrad 1 sowie 91.461 Sachleistungsbeziehende mit einem höheren Pflegegrad (Bayerisches Landesamt für Statistik 2021). Wenn etwa die Hälfte dieser Anspruchsberechtigten tatsächlich eine Pflegeberatung in Anspruch nähme, läge die Summe der obligatorischen und fakultativen Pflegeberatungen in Bayern bei schätzungsweise 380.000 im Jahr. In der hier untersuchten Stichprobe sind im selben Zeitraum 25.913 Pflegeberatungen erfasst. Das waren zwar „nur“ rund 7 % aller schätzungsweise im Jahr 2020 in Bayern durchgeführten Pflegeberatungen, aber da kein Faktor erkennbar ist, warum die auf einer Vollerhebung der BARMER Pflegekasse basierende Stichprobe in irgendeiner Weise systematisch verzerrt gewesen sein könnte, kann sie als repräsentativ für die Grundgesamtheit angesehen werden.

### Auswirkungen der Angst vor SARS-CoV-2 auf die Inanspruchnahme der Pflegeberatung

**Figure 1:**
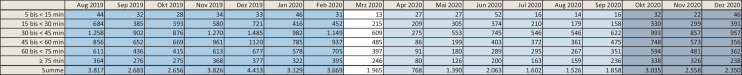

Die Häufigkeiten der von ambulanten Pflegediensten abgerechneten 37.3-Pflegeberatungen in den Monaten von August 2019 bis Dezember 2020 zeigt Abb. 1. Dargestellt sind zum einen die Gesamtzahl der Pflegeberatungen in den einzelnen Monaten, zum anderen die nach Klassen gruppierte Dauer der Pflegeberatungen (auf die später eingegangen wird).

**Figure 2:**
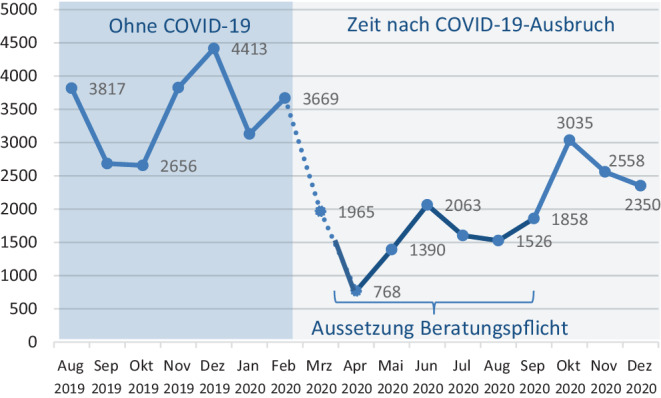

Die Abb. 2 visualisiert die bereits in Abb. 1 dargestellten monatlichen Beratungsmengen. Hinsichtlich der Anzahl der Pflegeberatungen (pro Monat) ist zunächst grob zwischen der Zeit vor und der Zeit nach dem COVID-19-Ausbruch zu unterscheiden. Eine zweite Unterscheidungsebene ergibt sich durch die Aussetzung der Beratungspflicht. Die Zeitspanne zwischen dem Beschluss zur Aussetzung der Beratungsplicht am 27. März (dass die Beratungspflicht rückwirkend ab Januar ausgesetzt wurde, ist im hier interessierenden Zusammenhang unerheblich) bis zu deren Ende am 30. September ist in der Abbildung optisch dunkler dargestellt. Die Zeitspanne nach Ausbruch der Pandemie und vor dem Beschluss zur Aufhebung der Beratungspflicht ist in der Abbildung als gestrichelte Linie dargestellt.

Durch Abb. 2 wird u. a. deutlich, dass die monatliche Beratungsmenge auch schon in den Zeit *vor* dem COVID-19-Ausbuch nicht unbeträchtlich schwankte. Die Gründe dafür sind nicht klar. Das Hoch im Dezember 2019 kann noch am ehesten damit erklärt werden, dass zum Ende des Jahres noch ausstehende Pflegeberatungen „abgehakt“ wurden. Was dann allerdings nach dem Ausbruch der COVID-19-Pandemie passierte, überstieg bei Weitem das Ausmaß saisonaler oder zufälliger Schwankungen. Die monatliche Anzahl abgerechneter Pflegeberatungen brach ein und erreichte im April 2020 mit 768 einen einmaligen Tiefstand. Das entsprach nur noch einem Fünftel (20,6 %) der noch im Februar 2020 durchgeführten Beratungsmenge.

Da während des gesamten Aprils (streng genommen: bereits seit dem 27. März) die Aussetzung der Beratungspflicht sowie die Sorge vor einer Ansteckung mit SARS-Cov-19 darin zusammenwirkten, die Nachfrage nach Pflegeberatungen zu reduzieren, war mit einem starken Rückgang der Nachfrage in dieser Zeit (im Vergleich zur Ära vor COVID-19) zu rechnen gewesen (Hypothese H1).

**Figure 3:**
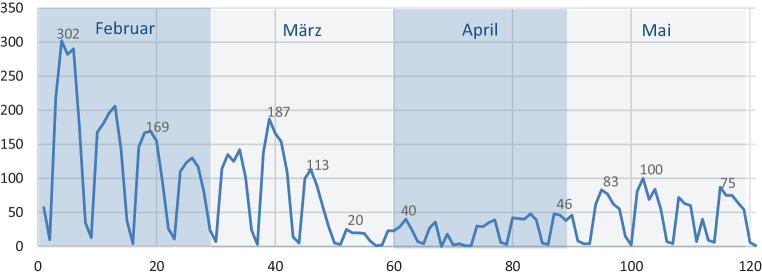

Der März verdient eine besondere Betrachtung, da die COVID-19-Epidemie *im Laufe *dieses Monats zum allgemeinen Lockdown und zu den gesetzlichen Ausnahmeregelungen bei der Pflegeberatung führte. Über die Entwicklung im März kann von daher nur die tageweise Betrachtung aus Abb. 3 Aufschluss geben.

**Figure 4:**
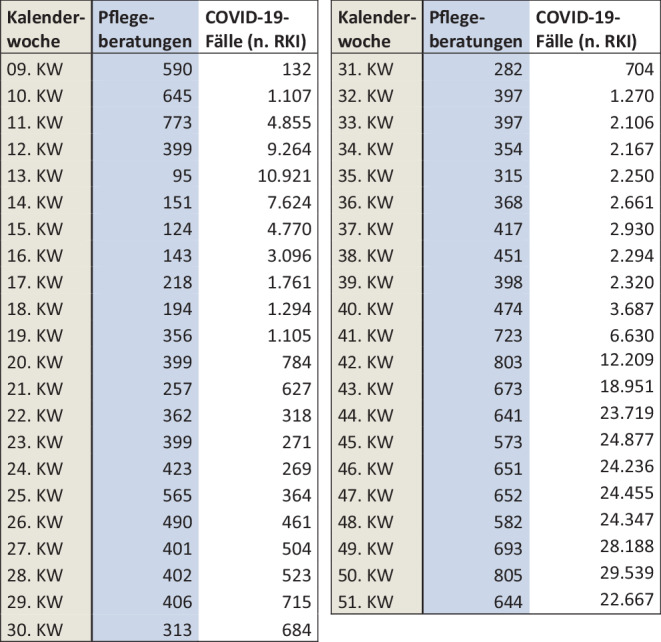

Die Abb. 3 zeigt zunächst Einbrüche im Wochenverlauf; diese lassen sich darauf zurückführen, dass an Wochenenden kaum Rechnungen gestellt werden; diese Schwankungen sind von daher zu vernachlässigen. Relevant ist hingegen der steile Abfall der Beratungszahlen im letzten Märzdrittel: Bereits in der Woche vor dem 27. März (dem Tag, als die Aussetzung der Beratungspflicht beschlossen wurde) sank die Anzahl der täglichen Pflegeberatungen von rund 100 auf rund 20 ab. In der am 23. März beginnenden 13. Kalenderwoche wurde der bisherige Tiefstand von 95 Beratungen/Woche gemessen (Abb. 4). Es steht zu vermuten, dass ein Großteil der bereits terminierten Beratungstermine aus Angst vor einer Infektion mit SARS-CoV‑2 abgesagt worden ist.

Die wöchentliche Anzahl der 37.3-Beratungseinsätze von der 9. Woche (ab 24. Februar) bis zum Ende des Jahres (51. Woche) korreliert stark (r = 0,567) mit den in diesen Wochen dem Robert Koch-Instituts gemeldeten COVID-10-Erkrankungen. Bei einem festgelegten Signifikanzniveau von 0,01 ist dieses Ergebnis mit einer Fehlerwahrscheinlichkeit von 0,000036 (*p*-Wert auf der Basis einer einseitigen Verteilung) hochsignifikant.

### Inwieweit führt die Aussetzung der Beratungspflicht zu einem Rückgang der Nachfrage nach 37.3-Pflegeberatungen?

Zur Beantwortung dieser Forschungsfrage (F2) muss die Wirkung des Faktors „Aussetzung der Beratungspflicht“ nach Möglichkeit von der Wirkung des Faktors „Angst vor einer Ansteckung mit SARS-CoV-2“ isoliert werden. Das Problem dabei ist, dass die Inanspruchnahme von Pflegeberatungen im fraglichen Zeitraum unterschiedlichen Einflüssen ausgesetzt gewesen ist. Bei Ausbruch der Pandemie war die Ansteckungsangst mit großer Wahrscheinlichkeit der alles überlagernde Grund für das Nichtzustandekommen von Pflegeberatungsgesprächen. Ab dem 27. März – aber in der Wirkung sicherlich verzögert – trat dann die Aussetzung der Beratungspflicht als zweiter Faktor hinzu. Die Wirkung des ersten Faktors wurde wenig später konterkariert durch einen dritten Faktor, nämlich durch den Faktor „Kenntnis der telefonischen Beratungsoption“. Denn für eine pflegebedürftige Person (oder eine Angehörige), die nicht mehr davon ausgeht, dass die Beratung wie gehabt in der eigenen Häuslichkeit erfolgen muss, kann das damit verbundene Ansteckungsrisiko objektiv kein Grund mehr sein, einen Beratungstermin abzusagen oder auszuschlagen.

**Figure 5:**
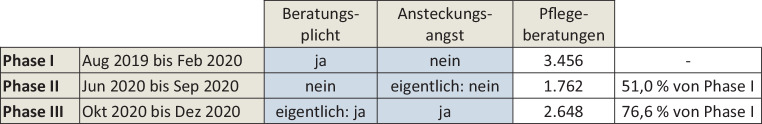

Wann wird die weit überwiegende Mehrheit der zu beratenden Personen Kenntnis von der telefonischen Beratungsoption erlangt haben? Ab Mitte April verbreitete sich unter den Pflegediensten die Information, dass eine Pflegeberatung auch per Telefon (oder online) möglich sei. Da die Pflegedienste aus ökonomischen Gründen ein Interesse daran haben, die Einnahmen aus ihren Pflegeberatungsleistungen auch tatsächlich zu generieren, kann unterstellt werden, dass die meisten Pflegedienste zumindest ihre Bestandskundinnen und -kunden zeitnah über die risikolose telefonische Beratungsoption informiert haben, sodass diese Information im Laufe des Monats Mai einen Großteil der infrage kommenden Pflegehaushalte erreicht haben sollte. Da in etwa zeitgleich dazu die erste Pandemiewelle ohnehin abebbte, kann angenommen werden, dass im Juni 2020 der Faktor „Angst vor einer Ansteckung mit SARS-CoV-2“ sowohl subjektiv als auch objektiv nur noch eine marginale Rolle spielte. Der Zeitraum von Juni bis zum Aussetzen der Beratungspflicht Ende September 2020 erscheint jedenfalls für die Beantwortung der Forschungsfrage (F2) prädestiniert. Nennen wir diesen Zeitraum: „Phase II“. Als Referenzzeiträume für Vergleiche mit Phase II bieten sich zwei andere Zeiträume an: nämlich zum einen die Monate August 2019 bis Februar 2020 (also *vor *dem Ausbruch der Pandemie liegende Monate = Phase I), zum anderen die Monate Oktober bis Dezember 2020, die zum einen von der zweiten Pandemiewelle und zum anderen von der Wiedereinführung der Beratungspflicht geprägt waren (= Phase III). In der Abb. 5 sind die kennzeichnenden Merkmale dieser drei Phasen übersichtlich dargestellt; ebenfalls dargestellt ist die Anzahl der Pflegeberatungen im monatlichen arithmetischen Mittel.

Wie aus Abb. 5 hervorgeht, fanden in den Monaten der Phase II kaum mehr als die Hälfte (51 %) der Pflegeberatungen statt, die in den 7 Monaten vor Ausbruch der COVID-19-Pandemie (Phase I) üblich gewesen waren (1762 statt 3456 Beratungen).

Da die Phase II (Jun 2020 bis Sep 2020) extra so definiert ist, dass der noch im März und April stark wirksame Faktor „Ansteckungsangst“ weder objektiv noch subjektiv eine Rolle spielen sollte, ist der Rückgang um 49 % im Vergleich zu Phase I weitgehend auf das Aussetzen der Beratungspflicht zurückzuführen. Zwar bleibt eine gewisse Unschärfe, da der Anteil derjenigen Beratungsberechtigten, die keine Kenntnis von der (risikolosen) telefonischen Beratungsoption hatten, unbekannt ist. Immerhin kann aber als Antwort auf Forschungsfrage F2 formuliert werden, dass die Aussetzung der Beratungspflicht zu einem massiven Rückgang der Nachfrage nach 37.3-Pflegeberatungen der Pflegeberatungen geführt hat.

Die Anzahl der Pflegeberatungen im Oktober 2020, dem ersten Monat nach der Wiedereinführung der Beratungspflicht, lag mit 3035 in etwa wieder auf dem Niveau vor dem Ausbruch von COVID-19 (Abb. 2). Die Werte für November und Oktober lagen aber wieder deutlich darunter, sodass der gemittelte Monatswert in Phase III (2648) insgesamt um 23,4 % unter dem gemittelten Monatswert von Phase I liegt. Hier machte sich offenkundig die im Zuge der zweiten Pandemiewelle wieder deutlich angestiegene Angst vor einer Ansteckung mit SARS-CoV‑2 bemerkbar.

Anzunehmen ist prima vista, dass der Beratungsmengenrückgang von Phase I zu Phase III v. a. auf den Verzicht fakultativer Beratungen zurückzuführen ist – schließlich kann auf obligatorische Beratungen per definitionem eigentlich gar nicht „verzichtet“ werden. Gegen diese Vermutung spricht allerdings, dass Pflegedienste 94 % ihrer fakultativen Pflegeberatungen (bei 61.461 von 97.591 Pflegeberatungsnutzerinnen und -nutzern; Bayerisches Landesamt für Statistik 2021) für „ihre“ Sachleistungskundinnen und -kunden durchführ(t)en. Und für jemanden, der für seine „alltägliche“ Versorgung Pflegekräfte in die Wohnung lassen (muss), ergäbe es kaum Sinn, aus Angst vor einer Infizierung mit SARS-CoV‑2 auf die vergleichsweise seltenen Pflegeberatungen zu verzichten. Das nach dieser Überlegung verbleibende Potenzial von nur noch 6 % fakultativer Pflegeberatungen ist aber viel zu klein, um die Differenz zwischen Phase I und Phase III erklären zu können. Von daher ist anzunehmen, dass nach der Wiedereinsetzung der Beratungspflicht längst nicht wieder alle Pflichtberatungen stattgefunden haben – jedenfalls nicht turnusgemäß. Tatsächlich musste die BARMER Pflegekasse vor dem Hintergrund der wieder eingesetzten zweiten Pandemiewelle unter Beachtung der geltenden Rahmenbedingungen nicht streng auf die Einhaltung der Fristen pochen.

### Dauer der Pflegeberatungen

**Figure 6:**
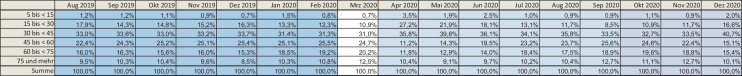

Da auf dem Nachweisformular über einen Beratungsbesuch nach § 37 Abs. 3 SGB XI (GKV-Spitzenverband o.J.) nicht anzukreuzen ist, ob ein Beratungsgespräch in Präsenz oder per Telefon erfolgt ist, kann die Forschungsfrage (F3) *„Sind telefonisch durchgeführte Pflegeberatungen im arithmetischen Mittel kürzer als Beratungen in der Häuslichkeit der zu beratenden Personen?“ *nur indirekt beantwortet werden. Die vorliegenden Daten zur Dauer der 37.3-Beratungseinsätze liefern immerhin Hinweise zur Beantwortung dieser Forschungsfrage. Die Abb. 6 basiert auf den bereits in Abb. 1 dargestellten Häufigkeiten.

Aus Abb. 6 geht u. a. hervor, dass zwar die meisten Pflegeberatungen zwischen 30 und < 45 min dauerten, dass aber die darüber liegenden Klassen („45 bis < 60 min“, „60 bis < 75 min“ und sogar „≥ 75 min“) ebenfalls stark besetzt waren. Besonders auffällig ist die im Vergleich zu den Vormonaten extreme Verkürzung der durchschnittlichen Beratungsdauer im April 2020. Der Anteil der Beratungen in der Klasse „15 bis < 30 min“ vergrößerte sich deutlich von 10,9 auf 27,2 %; spiegelbildlich dazu verringerten sich der Anteil der Klasse „45 bis < 60 min“ an den Beratungen sowie, etwas weniger deutlich, der Anteil der Klasse „60 bis < 75 min“.

**Figure 7:**
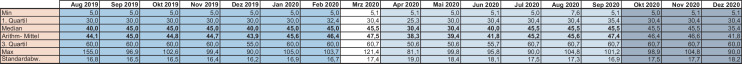

Weiteren Aufschluss über die Dauer der Pflegeberatungen gibt Abb. 7; hier ist die Entwicklung hinsichtlich der wichtigsten Lage- und Streuungsparameter dargestellt.

**Figure 8:**
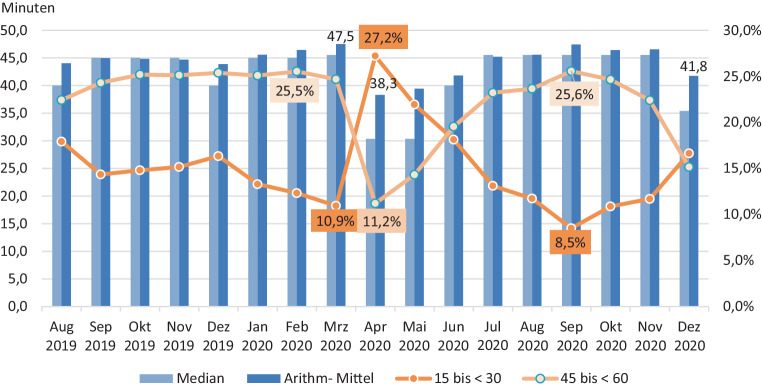

Die Abb. 8 kombiniert Parameter aus den Abb. 6 und 7; sie visualisiert also Veränderungen hinsichtlich der Dauer von Pflegeberatungen. Als Säulen dargestellt ist die Entwicklung der arithmetischen Mittelwerte und der Mediane aus Abb. 7. Als Linien dargestellt ist die in Abb. 6 beschriebene Entwicklung bei den Klassen „15 bis < 30 min“ sowie „45 bis < 60 min“ (auf die Abbildung der Entwicklung bei den anderen Klassen aus Abb. 6 wurde verzichtet, da die Abb. 8 sonst zu unübersichtlich geworden wäre).

Ins Auge springt in Abb. 8 die bereits angesprochene erhebliche Verschiebung von längeren zu kürzeren Pflegeberatungen im April 2020. Das arithmetische Mittel lag im April bei 38,3 min, nachdem es im Vormonat noch 47,5 min betragen hatte. Noch deutlicher, nämlich um rund ein Drittel, lag im April (und Mai) der Median unter den Medianen der Monate vor dem Pandemieausbruch.

Da im Laufe des Aprils 2020 ambulante Pflegedienste damit begannen, telefonische Beratungen durchzuführen, erscheint die deutliche Verkürzung der durchschnittlichen Beratungsdauer in diesem Monat prima vista als starke Unterstützung für die Hypothese H3, wonach telefonisch durchgeführte Pflegeberatungen im arithmetischen Mittel kürzer sind als Beratungen in der Häuslichkeit der zu beratenden Personen.

Nicht ohne Weiteres mit dieser Hypothese vereinbar ist allerdings, dass die mittlere Dauer einer Pflegeberatung nicht wenigstens auf diesem niedrigen Niveau verharrte oder sich sogar auf einem noch niedrigerem Niveau stabilisierte (schließlich bestand im betrachteten Zeitraum die Option telefonischer Pflegeberatungen). Tatsächlich dauerten Pflegeberatungen in den Monaten Juli bis November aber im arithmetischen Mittel wieder in etwa so lange wie in den Monaten vor der Ermöglichung telefonischer Pflegeberatungen (um im Dezember erneut abzusacken). Wie ist das zu erklären?

Hypothese H3 wäre nur dann widerlegt, wenn der Anteil derjenigen Pflegeberatungskunden, die eine telefonische Beratung präferieren (oder die mit einer solchen einverstanden wären), einigermaßen stabil wäre. Nur dann wäre damit zu rechnen gewesen, dass sich die durchschnittliche Beratungsdauer eines Beratungsgesprächs zwischen April und Juni immer weiter verkürzt hätte (korrelierend mit der Kenntnis der telefonischen Beratungsoption), um sich anschließend auf einem niedrigen Niveau einzupendeln.

Nun ist es ist zwar nicht unwahrscheinlich, dass sich der Anteil der per Telefon (oder online) durchgeführten Beratungen an der Gesamtmenge der Pflegeberatungen *nach* der Pandemie irgendwann stabilisieren könnte. Aber dafür, dass der Anteil der telefonischen Beratungen bereits in den fraglichen Monaten im Wesentlichen gleich geblieben ist, spricht nichts. Viel wahrscheinlicher ist es, dass der in diesem Zeitraum höchst volatile Faktor „Angst vor einer Infizierung mit SARS-CoV-2“ Einfluss auf den Anteil der Telefonberatungen an allen Pflegeberatungsgesprächen hatte; und zwar dergestalt, dass dieser Anteil ebenfalls schwankte. Nahm die Angst vor einer Infizierung mit SARS-CoV‑2 zu, verschob sich das Verhältnis von (im arithm. Mittel längeren) Präsenzberatungen zu (im arithm. Mittel kürzeren) Telefonberatungen zugunsten Letzterer.

Die im Vergleich in den Folgemonaten unerwartet kurze durchschnittliche Beratungsdauer im April ist demnach wahrscheinlich darauf zurückzuführen, dass in diesem Monat (dem Höhepunkt der ersten Pandemiewelle) besonders viele Pflegeberatungen telefonisch durchgeführt worden sind. Zwar wussten im April weniger Pflegedienste von dieser Option als im Mai (oder noch später), aber wer von ihr Kenntnis hatte, machte davon aus „Coronaangst“ wahrscheinlich eher Gebrauch als in späteren Monaten. Schließlich ließ ab Mai (mit dem Abebben der 1. Coronawelle) auch die Sorge vor einer Ansteckung mit SARS-CoV‑2 nach. Zumal als weiterer Faktor hinzukommt, dass in den Sommermonaten die Wetterlage eher ein Gespräch auf dem Balkon oder der Terrasse zuließ als im Vorfrühling oder im Herbst/Winter. Wie Abb. 8 verdeutlicht, weist die Kurve der Klasse „45 bis < 60 min“ bis einschließlich September nach unten und die Kurve der Klasse „15 bis < 30 min“ nach oben. Mit Beginn der zweiten Pandemiewelle am Ende des Sommers kippte diese Entwicklung wieder in ihr Gegenteil. Auch die durchschnittliche Beratungsdauer begann sich wieder zu verkürzen. Im Dezember (also auf dem Höhepunkt der zweiten Pandemiewelle; RKI 2021a) lagen die Parameterwerte für die Dauer von Pflegeberatungen zwar noch nicht wieder auf dem Niveau des Coronaschocks im April, aber sie hatten sich diesem Niveau deutlich angenähert. Sehr wahrscheinlich ist, dass in dieser Zeit wieder vermehrt Telefonberatungen durchgeführt wurden, die eben im Durchschnitt kürzer waren als Präsenzberatungen.

Andere mögliche Erklärungen können nicht überzeugen. So ist zwar theoretisch nicht auszuschließen, dass Beratungsnutzende aus Furcht vor einer Ansteckung die Kontaktzeit einer Präsenzberatung (und damit die Viruslast in der Raumluft) verkürzt haben, ein solches Verhalten widerspräche aber allen Erfahrungen (wer sich sehr vor einer Infektion fürchtete, wird sich in aller Regel erst gar nicht auf ein Präsenzberatungsgespräch eingelassen haben). Etwas wahrscheinlicher ist es da schon, dass das eine oder andere Präsenzberatungsgespräch von den Pflegefachkräften aus Ansteckungsangst abgekürzt wurde, wenn sie in einem Pflegehaushalt merkten, dass dort die Hygienestandards in keinster Weise eingehalten wurden.

### Auswirkungen von Art und Höhe der Vergütungen

Zur Prüfung der mit F4 verbundenen Hypothese H4.1 *(Die durchschnittliche Dauer eines 37.3-Pflegeberatungsbesuchs war in den Monaten August 2019 bis Februar 2020 deutlich länger als in den Monaten vor der Einführung des neuen Abrechnungssystems in Bayern)* bzw. Hypothese H4.2 *(In den Monaten seit dem Pandemieausbruch hat sich zwar die durchschnittliche Dauer eines 37.3-Pflegeberatungsbesuchs verglichen mit Monaten August 2019 bis Februar 2020 verkürzt, sie ist aber immer noch länger als vor der Einführung des neuen Abrechnungssystems in Bayern)* fehlen „belastbare“ Zahlen für die Zeit *vor* der Einführung des neuen Abrechnungssystems in 2019.

Valide sind immerhin die aus den Abrechnungsdaten der BARMER Pflegekasse in Bayern ermittelten Zahlen:

- In der Zeitspanne von August 2019 bis Februar 2020 wurden von den ambulanten Diensten in Bayern für einen Beratungseinsatz im arithmetischen Mittel 44,9 min veranschlagt.
- In der Zeitspanne von April bis Dezember 2020 wurden im arithmetischen Mittel 43,6 min veranschlagt.

Als Referenz für diese aus den Abrechnungsdaten ermittelten Zahlen muss auf die bereits erwähnten, über 10 Jahre alten Ergebnisse der Befragung von lediglich 25 Pflegediensten aus Nordrhein-Westfalen durch Büscher et al. (2010) zurückgegriffen werden. Arithmetische Mittelwerte waren damals: 26 min in Pflegestufe I, 30 min in Pflegestufe II und 35 min in Pflegestufe III (Büscher et al. 2010, S. 24).

Die Vergleichbarkeit dieser Zahlen mit den oben genannten wird zwar nicht nur durch Zeit (2009/2010 vs. 2019/2020) und Raum (NRW vs. Bayern) eingeschränkt, sondern auch durch die unterschiedliche Datengrundlage (Befragungsergebnisse vs. Abrechnungsdaten). Bei aller gebotenen Vorsicht kann dennoch festgehalten werden, dass sowohl die Hypothese H4.1 als auch die Hypothese H4.2 durch die verfügbaren Daten und Informationen gestützt werden.

Das bayerische Abrechnungssystem nimmt von den Pflegeberaterinnen und -beratern den ökonomischen Druck, eine Beratung in möglichst kurzer Zeit durchzuführen. Vielmehr bietet das Abrechnungssystem einen wirtschaftlichen Anreiz, Pflegeberatungsgespräche bis zur abrechnungsfähigen Höchstdauer von 75 min auszudehnen. Tatsächlich dauerte etwa jede zehnte Pflegeberatung (9,79 %) ziemlich exakt 75 min, während nur 0,33 % der Pflegeberatungen darüber hinausgingen (mindestens 5 min länger als 75 min).

## Schlussfolgerungen

Mit der vorliegenden Studie wird der – beträchtliche – Einfluss der COVID-19-Pandemie auf Anzahl und Dauer der von ambulanten Pflegediensten erbrachten Pflegeberatungen nach § 37 Abs. 3 SGB XI erstmals quantifiziert. Es besteht eine deutliche Korrelation zwischen den COVID-19-Fällen und der Anzahl der Beratungsbesuche; die Angst vor einer Ansteckung mit SARS-CoV‑2 hat sich insbesondere in der ersten Pandemiewelle massiv reduzierend auf die Inanspruchnahme von Präsenzberatungen in der eigenen Häuslichkeit ausgewirkt.

Als Reaktion auf die COVID-19-Pandemie hat der Gesetzgeber zum einen die Beratungspflicht – vorübergehend – ausgesetzt, zum anderen die Möglichkeit telefonischer und online durchgeführter Beratungen geschaffen. Da beide Maßnahmen denkbare Optionen für die Zeit „nach Corona“ sind, galt ein Hauptaugenmerk der Studie deren Analyse – wobei der Faktor „Ansteckungsangst“ in diesem Zusammenhang eine Störvariable war.

Die vorliegenden Daten deuten darauf hin, dass ohne Beratungspflicht fast jede zweite Pflegeberatung wegfallen könnte. Der Geltungsbereich dieser Aussagen erstreckt sich zwar zunächst nur auf Bayern, allerdings ist kein Grund ersichtlich, warum sich die Beratungsanspruchsberechtigten in den anderen Bundesländern anders verhalten sollten.

Ein bayerisches Spezifikum stellt hingegen die Abrechnung der Pflegeberatungsbesuche nach Zeit dar. Da diese Abrechnungsform einen Anreiz für die Pflegedienste schafft, Pflegeberatungen zeitlich auszudehnen – gleich, ob in Präsenz oder per Telefon/digital –, sind die in dieser Studie getroffenen Aussagen zur Dauer von Pflegeberatungen nicht oder nur mit allergrößter Vorsicht auf andere Bundesländer übertragbar.

Wahrscheinlich ist, dass die Angst vor einer SARS-CoV-2-Infektion zu einer Verdrängung von Präsenzberatungen durch Telefonberatungen geführt hat. Wenn dies zutrifft, belegen die vorliegenden Daten, dass – selbst in Bayern (so muss man wohl konstatieren) – telefonische Pflegeberatungen im arithm. Mittel kürzer sind als Präsenzberatungen.

Gemessen daran, dass die von ambulanten Diensten durchgeführten Pflegeberatungen nach § 37 Abs. 3 SGB XI von großer Relevanz für die Sicherstellung der häuslichen Pflege pflegebedürftiger Menschen sind, liegen über dieses Feld überraschend wenige wissenschaftlich gesicherte Erkenntnisse vor (Hallensleben 2021). Die vorliegende Studie hat diese Lücke nur zu einem kleinen Teil schließen können.

Größtes Desiderat wäre eine Dokumentenanalyse der von den ambulanten Pflegediensten ausgefüllten Beratungsprotokolle. Würden wenigstens die quantitativen Items des Formular „Nachweis über einen Beratungsbesuch nach § 37 Abs. 3 SGB XI“ regelhaft erfasst, hätte sich der in dieser Studie eingeschlagene Umweg über die Abrechnungsdaten erübrigt.
